# Enhanced Hydrogenation Performance over Hollow Structured Co‐CoO*x*@N‐C Capsules

**DOI:** 10.1002/advs.201900807

**Published:** 2019-09-03

**Authors:** Hao Tian, Xiaoyan Liu, Liubing Dong, Xiaomin Ren, Hao Liu, Cameron Alexander Hurd Price, Ying Li, Guoxiu Wang, Qihua Yang, Jian Liu

**Affiliations:** ^1^ State Key Laboratory of Catalysis iChEM Dalian Institute of Chemical Physics Chinese Academy of Sciences 457 Zhongshan Road Dalian 116023 China; ^2^ Department of Chemical Engineering Curtin University Perth WA 6845 Australia; ^3^ Centre for Clean Energy Technology School of Mathematical and Physical Sciences Faculty of Science University of Technology Sydney Broadway Sydney NSW 2007 Australia; ^4^ Institute of Industrial Catalysis Zhejiang University of Technology 18 Hangzhou Chaowang Road Hangzhou 310032 China; ^5^ Joint International Laboratory on Environmental and Energy Frontier Materials School of Environmental and Chemical Engineering Shanghai University Shanghai 200444 China; ^6^ DICP‐Surrey Joint Centre for Future Materials Department of Chemical and Process Engineering and Advanced Technology Institute University of Surrey Guilford Surrey GU2 7XH UK

**Keywords:** confined catalysis, hydrogenation, mesoporous materials, nanoreactors

## Abstract

It is desirable to design nonprecious metal nanocatalysts with high stability and catalytic performance for fine chemicals production. Here, a method is reported for the preparation of cobalt metal and cobalt oxide cores confined within nanoporous nitrogen‐doped hollow carbon capsules. Core–shell structured Zn/Co‐ZIF@polymer materials are fabricated through a facile coating polymer strategy on the surface of zeolitic imidazolate frameworks (ZIF). A series of hollow carbon capsules with cobalt metal and cobalt oxide are derived from a facile confined pyrolysis of Zn/Co‐ZIF@polymer. The hollow Co‐CoO*x*@N‐C capsules can prevent sintering and agglomeration of the cobalt nanoparticles and the nanoporous shell allows for efficient mass transport. The specific surface area and Co particle size are optimized through finely tuning the original Zn content in ZIF particles, thus enhancing overall catalytic activity. The yolk–shell structured Zn_4_Co_1_O_x_@carbon hollow capsules are shown to be a highly active and selective catalyst (selectivity >99%) for hydrogenation of nitrobenzene to aniline. Furthermore, Zn_4_Co_1_O_x_@carbon hollow particles show superior catalytic stability, and no deactivation after 8 cycles of reaction. The hollow Co‐CoO*x*@N‐C capsules may shed light on a green and sustainable catalytic process for fine chemicals production.

## Graphical abstract

**Cobalt metal and cobalt oxide cores confined within nanoporous nitrogen‐doped hollow carbon capsules** are achieved. The hollow Co‐CoO*x*@N‐C capsules exhibit high catalytic activity and selectivity in hydrogenation of nitrobenzene to aniline.

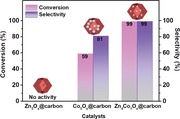

## 1 Introduction

Catalytic hydrogenation of nitroarenes to their substituted anilines are of importance not only for academic research but for chemical industry such as the production of dyes, pharmaceuticals, pigments, and agrochemicals.^1^, ^2^ Noble‐metal catalysts have been proved to be very efficient for catalytic hydrogenation reactions.^3^, ^4^, ^5^, ^6^, ^7^, ^8^ However, these noble metals are expensive and rare. Therefore, transitional metals that are cheap, abundant, and capable of promoting catalytic activity have received considerable attention. For example, iron and cobalt catalysts have also been demonstrated to facilitate the highly selective hydrogenation of nitroarenes.^2^, ^9^, ^10^, ^11^, ^12^ However, it is still challenging to explore relatively cheap transitional metal catalysts for the hydrogenation of nitroarenes with high catalytic activity and chemoselectivity. Furthermore, it is an ideal model system to explore the fundamental catalytic phenomena such as cooperative catalysis, isolated effect, and tandem catalysis in confined space.

Nanoporous carbon materials have extraordinary properties due largely to excellent electrical conductivity, high surface area, chemical inertness, mesoporosity, and good biocompatibility.^13^, ^14^, ^15^, ^16^ These materials have been widely investigated because of their applications in adsorption, catalysis, energy storage, and conversion.^17^, ^18^, ^19^, ^20^ Based on the previous research, composition, porosity adjustment, morphology control, doping effect, graphitization, and surface modification can contribute to the improvement of both electrochemical and catalytic performance of a material. Thus, when considering porous structures, nanoporous hollow carbons have received considerable attention^21^, ^22^, ^23^, ^24^, ^25^, ^26^, ^27^ due to several desirable advantages: 1) the hollow interior provides a homogeneous reaction environment for heterogeneous catalysis, 2) protection and separation of the catalytic core nanoparticles to prevent sintering and coking, and 3) controllable diffusion rates. So far, catalytic nanoparticles including noble metals and metal oxides have been successfully incorporated into nanoporous shells with various compositions by the template method.^26^, ^27^, ^28^, ^29^ However, it still remains a great challenge to confine catalytic nanoparticles in hollow capsules with complicated architecture and compositions at the nanoscale, owing to the delicacy required in controlling chemical reactions and materials compatibility within the encapsulated hollow shells. Therefore, it is still necessary to achieve the fundamental understanding of the catalytic process inside a confined space in hollow‐structured reactor with cobalt particles inside it.

Metal–organic frameworks (MOFs) have been widely used as precursors for constructing nanostructured carbon composites due to their unique thermal behavior and chemical reactivity.^18^, ^19^, ^30^, ^31^, ^32^, ^33^, ^34^, ^35^, ^36^, ^37^ For example, we reported the synthesis of yolk‐shell structured sub‐microreactors with loaded metal nanoparticles into ZnO‐microporous carbon core–shell structures.^37^ When used as catalysts for hydrogenation of phenylacetylene to phenylethylene, the prepared Pd&ZnO@carbon sub‐microreactor showed higher conversion and selectivity than that of Pd/ZnO and Pd/C with similar Pd loading. High selectivity (99%) and superior catalytic stability, and no deactivation after 25 h of reaction were presented by using this catalyst. However, it is still difficult to overcome the sintering and aggregation of transitional‐metal nanoparticles during calcination conditions, which have detrimental effects on catalytic activity.^38^ Recently, it has been reported that Zn‐based MOFs can be synthesized and zinc species can be removed because of the evaporation of Zn and associated evolution of CO*_x_* species through calcination in an inert atmosphere.^39^, ^40^ Furthermore, it has also been found that the introduction of inactive Zn nanoparticles can suppress the sintering of the remaining active transitional species and enhance their dispersion in the carbon structures, further improving their electrocatalytic activities.^41^

Herein, inspired by the well‐established carbon spheres library and its related coating technique,^13^, ^42^, ^43^, ^44^, ^45^, ^46^, ^47^, ^48^, ^49^ a new methodology for the preparation of monometallic/metal oxide confined within nitrogen‐doped hollow carbon capsules is presented. The hollow void between monometallic/metal oxide and carbon shell can provide a homogeneous chemical microenvironment for hydrogenation catalytic reaction. The sintering of cobalt particles can be suppressed, and the specific surface area of the remaining catalysts is increased because of the spatial isolation of Zn species and the high volatility at high temperature. The outer carbon shell prevents the catalytically active Co nanoparticles from agglomeration and coking. The specific surface area, Co nanoparticle size, and hence catalytic activity can be optimized through finely tuning the original Zn content in ZIF particles. The catalytic performance of these Co@C hollow capsules is evaluated by the hydrogenation nitroarenes to their substituted anilines.

## 2 Results and Discussions

**Scheme 1:**
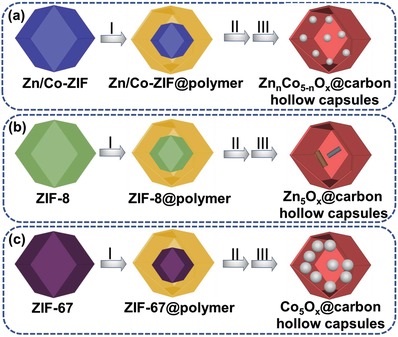
Schematic illustration of the fabrication of Zn*_n_*Co_5−_ *_n_*O*_x_*@carbon hollow capsules a): n_Zn/Co_ = 4 and 1; b): n_Zn/Co_ = 5; c): n_Zn/Co_ = 0)): I) uniform polymer coating process; II) template removal by hydrothermal treatment; III) formation of Zn*_n_*Co_5−_ *_n_*O*_x_*@carbon hollow capsules through annealing in N_2_ atmosphere. (Blue, green, purple, yellow, gray, brown, and dark red colors refer to Zn/Co‐ZIF, ZIF‐8, ZIF‐67, polymer layer, cobalt particles, ZnO particles, and carbon layer, respectively.

The synthesis strategy of Zn*_n_*Co_5−_ *_n_*O*_x_*@carbon hollow capsules is shown in **Scheme** 1. In the first step, the surface of ZIFs nanoparticles is coated with polymer layer by using the extended Stöber coating method to produce highly uniform core‐shelled ZIF@polymer core–shell structures. Subsequently, hydrothermal treatment was used to prepare yolk‐shell structured Zn*_n_*Co_5−_ *_n_*O*_x_*@polymer particles. Carbonization of these yolk‐shell structured particles in nitrogen atmosphere generated Zn*_n_*Co_5−_ *_n_*O*_x_*@carbon hollow capsules. Scheme 1a illustrates that through direct pyrolysis of Zn/Co‐ZIF@polymer precursor, small and well‐dispersed cobalt particles within nitrogen‐doped carbon shells can be obtained, which shall be referred to as Zn*_n_*Co_5−_ *_n_*O*_x_*@carbon particles. In addition, the porous nitrogen‐doped carbon shell and the formed yolk‐shell structures promotes mass transfer and prevents the catalytic active Co nanoparticles from several deactivation routes, i.e., sintering and coking, due to the spatial confinement effect. Through adjusting the original zinc content in the raw ZIF particles, the optimal specific surface area, Co nanoparticle size, and hence catalytic activity can be achieved. The unique characters of this novel material allow for superior catalytic activities in hydrogenation of nitrobenzene, as the addition of Zn species serves to enhance the dispersion of the Co nanoparticles and the interactions between the Co and the N‐doped carbon support. When ZIF‐8 particles were used as precursors (Scheme 1b), no catalytic activity was found when Zn_5_O*_x_*@carbon particles were exposed to the hydrogenation of nitrobenzene. Following the application of the purely ZIF‐67 based material (Scheme 1c) within the reaction, it was found that the catalyst presented low surface area and considerable agglomeration of cobalt nanoparticles with low catalytic activity were found.

**Figure 1:**
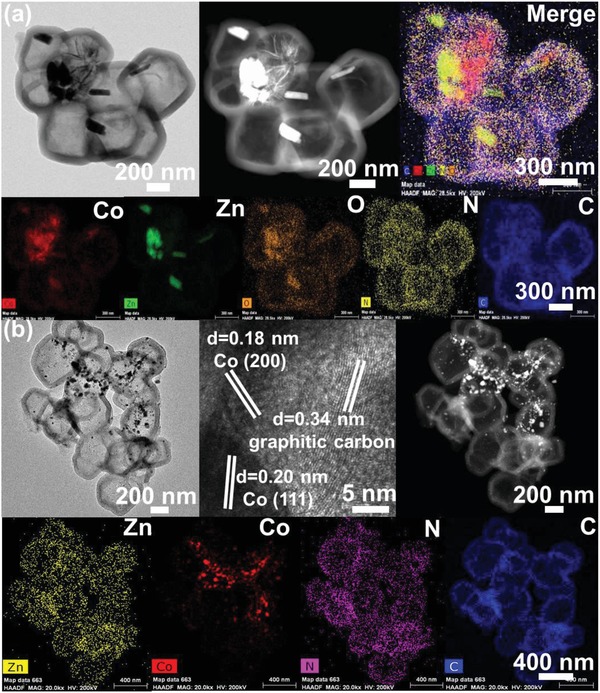
a) TEM image, HAADF image, STEM element mapping images of yolk‐shelled Zn_4_Co_1_O*_x_*@polymer. b) TEM images, HRTEM image, HAADF image, STEM element mapping images of Zn_4_Co_1_O*_x_*@carbon hollow capsules.

The production of each material was verified through analysis of the obtained X‐ray diffraction (XRD) patterns of ZIF‐8, ZIF‐67, and bimetallic ZIFs (Zn*_n_*Co_5−_ *_n_*‐ZIF) nanoparticles (Figure S1, Supporting Information), which agree well with previous reports^50^ and scanning electron microscope (SEM) images (Figure S2, Supporting Information) confirmed a rough surfaced polyhedral structure. 3‐aminophenol‐formaldehyde (APF) polymer was used as the coating layer for all the ZIFs particles, which later achieved the N‐doped carbon shells present in each variation. Polyvinylpyrrolidone (PVP) plays an important role for achieving the homogeneous coating of polymer on the surface of ZIF particles. After the negatively charged ZIF particles are covered with PVP molecules, polymerization sites can be provided to improve electrostatic forces for further uniformly adsorbing 3‐aminophenol and formaldehyde molecules. Huo and co‐workers reported that the presence of PVP molecules can assist the synthesis of metal nanoparticles@ZIF‐8 core–shell structures.^51^ It was reported that the strong coordination interactions between the terminal groups from PVP molecules and zinc metals assured the growth of ZIF particles. At the same time, PVP molecules can improve the dispersion of ZIF particles, which can increase the nucleation site of polymer. On this basis, uniform polymer layer can be successfully formed on the surface of ZIF particles. **Figure** 1a shows that further hydrothermal treatment for 24 h of core–shell structured Zn_4_Co_1_‐ZIF@polymer nanoparticles induced the generation of yolk‐shell structured materials with average shell thickness about 95 nm. It can be seen in these images that nanoribbons and needle‐like structures were formed within the hollow polymer shell. The scanning transmission electron microscopy (STEM) and the corresponding energy dispersive X‐ray spectroscopy (EDS) elemental mapping (Figure 1a) identified the uniform distribution of nitrogen, carbon, zinc, and oxygen in this hollow structure. Elemental carbon and nitrogen are homogenously distributed in the carbon shell, but Zn and Co atoms are dispersed uniformly throughout the nanoribbon and needle‐like nanoparticles, confirming elemental zinc and cobalt are confined inside the hollow polymer shell. In our recent work,^37^ we found that the structural evolution from core–shell structured ZIF‐8@polymer to yolk‐shell structured particles occurred due to the dissolution of ZIF‐8 and formation of the intermediate needle‐like particles. In this work, the possible formation mechanism should be similar for that case. Specifically, for the bimetallic Zn_4_Co_1_‐ZIF particles, the polymer on the surface of ZIF particles can result in the decomposition of Zn_4_Co_1_‐ZIF particles. Then the generation of ZnO nanoribbon and needle‐like cobalt particles can be observed. Pyrolysis of yolk‐shelled Zn_4_Co_1_O*_x_*@polymer at 700 °C under N_2_ atmosphere led to volatilization and carbonization of the organic constituents and reduction of the zinc and cobalt nanoparticles. Figure 1b illustrates that both nanoribbon and needle‐like structures in yolk‐shelled Zn_4_Co_1_O*_x_*@polymer disappeared and only small nanoparticles can be found with the carbon capsules with average shell thickness about 64 nm, indicating ZnO nanoribbons have been reduced by carbon frameworks and then volatilized during carbonization process.^37^ This can also be confirmed by SEM images in Figure S3a,b of the Supporting Information, STEM images, and element mapping results in Figure 1b. As shown in HRTEM image in Figure 1b, tiny Co nanoparticles were wrapped in the graphitic carbon layers and the interlayer distance of 0.34 nm is determined, corresponding to the (002) plane of graphitic carbon.^52^ The clear lattice fringes with *d*‐spacing of ≈0.18 and 0.20 nm are also displayed, which is attributed to the (200) and (111) lattice plane of cubic Co metal, indicating a high degree of crystallinity.

**Figure 2:**
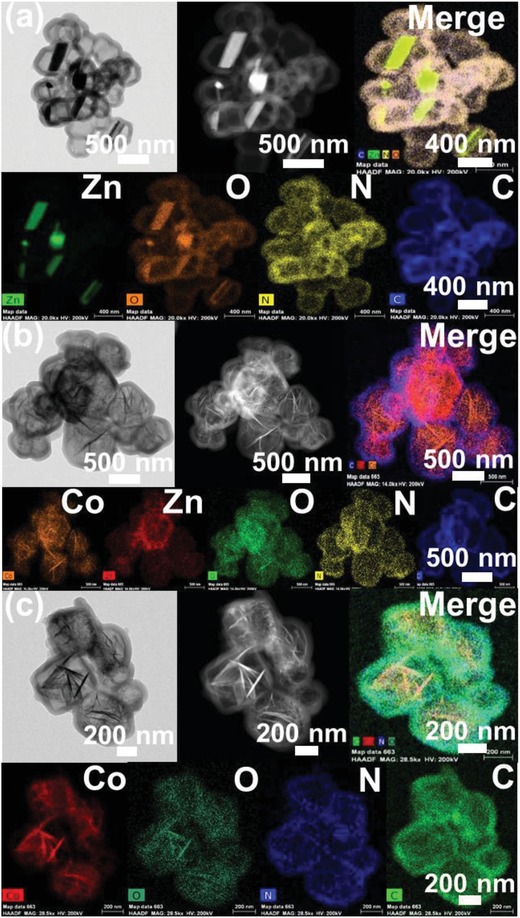
TEM images, HAADF images, STEM element mapping images of yolk‐shelled Zn*_n_*Co_5−_ *_n_*O*_x_*@polymer. a) n_Zn/Co_ = 5, b) n_Zn/Co_ = 1, and c) n_Zn/Co_ = 0.

After changing the molar ratio between Zn and Co to 5:0, only nanoribbon structures are generated within polymer shell with shell thickness about 105 nm, as shown in **Figure** 2a. STEM elemental mapping images in Figure 2a show that the Zn and O atoms are dispersed uniformly throughout the nanoribbon nanoparticles. Compared with Zn_4_Co_1_O@polymer nanoparticles, the introduction of cobalt ions into Zn_4_Co_1_‐ZIF frameworks induced the generation of needle‐like cobalt nanoparticles. Mapping of the carbon and nitrogen atoms of the capsules confirms their nitrogen‐doped carbon character. Interestingly, when decreasing the molar ratio of Zn and Co to 1:4, the nanoribbon structure disappears entirely from Figure 2b. Elemental mapping of yolk‐shell structured Zn_1_Co_4_O*_x_*@polymer indicates that elemental Zn and Co are dispersed uniformly on the inside nanoparticles within the hollow polymer shell. The transmission electron microscope (TEM) images of Co_5_O*_x_*@polymer in Figure 2c illustrates the absence of nanoribbon structures and only needle‐like nanostructures can be found in the polymer shell. The STEM image and elemental mapping in Figure 2c suggest Co atoms are dispersed uniformly throughout the needle‐like nanoparticles. Additionally, we investigated the effect of varying the polymer shell thickness, results of which can be seen in Figure S4 of the Supporting Information, for all four variants. This was achieved through simply controlling the amounts of the 3‐aminophenol and formaldehyde.

**Figure 3:**
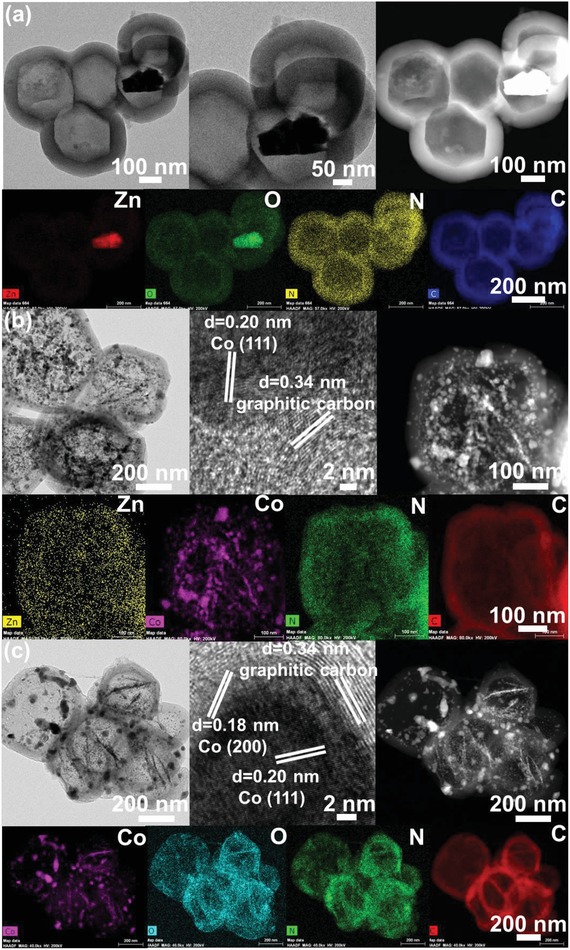
TEM images, HRTEM images, HAADF images, STEM element mapping images of yolk‐shelled Zn*_n_*Co_5−_ *_n_*O*_x_*@carbon hollow capsules. a) n_Zn/Co_ = 5, b) n_Zn/Co_ = 1, and c) n_Zn/Co_ = 0.

The above synthesized yolk‐shelled Zn*_n_*Co_5‐_ *_n_*O*_x_*@polymer nanoparticles (*n*_Zn/Co_ = 5, 1, and 0) were also calcined at 700 °C under N_2_ atmosphere to achieve carbon particles. As presented the SEM images (Figure S3c,d, Supporting Information) and TEM images in **Figure** 3a, yolk‐shell structured ZnO@carbon with decreased shell thickness about 70 nm are achieved and the nanoribbons have been changed into small ZnO particles within the carbon shell. Elemental mapping of yolk‐shell structured ZnO@carbon in Figure 3a show that zinc atoms are confined in the hollow carbon shell. When decreasing the molar ratio of Zn and Co to 1:4, Figure 3b shows that sintering of the nanoparticles into large aggregates was evident, and all the nanoparticles were observed within the interior of the carbon shell. The formation of hollow void of Zn_1_Co_4_O*_x_*@carbon particles is also confirmed from the SEM images in Figure S3e,f (Supporting Information). The average diameter of the cobalt nanoparticles in yolk‐shelled Zn_1_Co_4_O*_x_*@carbon hollow capsules is ≈24 nm and shell thickness has also declined to 47 nm (Figure 3b). This indicates that higher concentration of cobalt metal induces formation of larger cobalt nanoparticles. It can also be revealed that Co_5_O*_x_*@carbon hollow capsules with the highest cobalt concentration among those samples produced has largest average size of the cobalt nanoparticles viz., 28 nm (Figure 3c). Therefore, in Zn*_n_*Co_5−_ *_n_*O*_x_*@carbon hollow capsules with higher Zn content, the Co species are spatially isolated by dispersed Zn species and the samples with lower Zn contents lead to severe aggregation of cobalt nanoparticles. These results clearly demonstrate that the sintering of cobalt nanoparticles during carbonization can be effectively suppressed with the presence of Zn species at high contents due to spatial isolation of Co by the supporting Zn species. In addition, after decreasing the amount of 3‐aminophenol and formaldehyde (Figure S5, Supporting Information), partially hollow carbon capsules have been collapsed, indicating a minimum requirement of monomer and linking agent is required for successful formation of the polymer shell.

The compositions of yolk‐shell structured Zn*_n_*Co_5−_ *_n_*O*_x_*@carbon hollow capsules are shown in the XRD patterns (Figure S6, Supporting Information). Zn_5_Co_0_O*_x_*@carbon includes wurtzite phase ZnO (JCPDS#36‐1451). After decreasing the molar ratio between Zn and Co to 4, 1, and 0, characteristic peaks of ZnO are not detected from XRD patterns (Figure S6, Supporting Information). The newly formed peaks at 44.3°, 51.6°, and 76.1° can be ascribed to cubic cobalt metal (JCPDS#15‐0806) and other peaks at 36.8°, 42.7°, and 62.3° can be attributed to CoO (JCPDS#48‐1719). By using the Scherrer equation for the cobalt (111) peak in the XRD patterns, approximate cobalt particle size was calculated to be 11, 13, 24, and 28 nm with increasing cobalt content. This indicates that the samples with lower Zn contents resulted in severe Co aggregation.

Further structural information concerning our yolk‐shelled Zn*_n_*Co_5−_ *_n_*O*_x_*@carbon hollow capsules was provided through Raman spectroscopy analysis. As shown in Figure S7 of the Supporting Information, two peaks at about 1350 and 1592 cm^−1^are displayed in all samples, which are assigned to D and G bands, respectively. It is commonly believed that the D‐band is related to structural defects within the carbon frameworks. The intensity ratio of the D‐band and G‐band (*I*_D_/*I*_G_) is related to the amounts of defects in carbon nanoparticles derived from the loss of atoms through decomposition of oxygen‐containing groups during calcination process.^53^ The calculated *I*_D_/*I*_G_ intensity ratio of Zn_4_Co_1_O*_x_*@carbon (1.1) is higher than that of Zn_1_Co_4_O*_x_*@carbon (1.0) and Co_5_O*_x_*@carbon(1.0), indicative of more defects within the as‐made sample matrix.

The surface areas of those as‐prepared nanoparticles were determined by nitrogen sorption measurements, and the results are shown in Figure S8 of the Supporting Information and **Table** 1. As shown in Figure S8 of the Supporting Information, the nitrogen adsorption isotherms of the four samples were type IV with a distinct H3 hysteresis loop.^49^ As presented in Table 1, the Brunauer–Emmett‐Teller (BET) surface area and the pore volume obtained on the basis of the adsorption isotherm for ZnO@carbon hollow capsules were 407 m^2^ g^−1^ and 0.31 cm^3^ g^−1^, respectively. After introduction of cobalt nanoparticles into carbon capsules, the BET surface area and pore volume increased, respectively, 562 m^2^ g^−1^ and 0.61 cm^3^ g^−1^ for Zn_4_Co_1_O*_x_*@carbon, 465 m^2^ g^−1^ and 0.60 cm^3^ g^−1^ for Zn_1_Co_4_O*_x_*@carbon, 435 m^2^ g^−1^ and 0.60 cm^3^ g^−1^ for Co_5_O*_x_*@carbon, respectively. This tendency demonstrated that the specific surface area is associated with the compositional Zn/Co ratio and Zn*_n_*Co_5−_ *_n_*O*_x_*@carbon particles with different content of Zn could not only control the cobalt nanoparticle size but also regulate the specific surface area of the final products. It also indicates that the as‐prepared Zn*_n_*Co_5−_ *_n_*O*_x_*@carbon yolk‐shell particles possess abundant porous channels ensuring high permeation and mass transfer rates for species involved in a catalytic reaction.

**Table 1:** Physical properties of Zn*_n_*Co_5−_ *_n_*O*_x_*@carbon capsules

Zn*_n_*Co_5−_ *_n_*O*_x_*@carbon capsules	BET surface area [m^2^ g^−1^]a)	Pore volume [cm^3^ g^−1^]a)	Average cobalt particle size [nm]b)	Average shell thickness [nm]c)	*I*_D_/*I*_G_ d)	Co [wt%]e)	Zn [wt%]e)
*n* = 5	407	0.31	–	70	–	0	4.12
*n* = 4	562	0.61	11	64	1.08	4.81	0.36
*n* = 1	465	0.60	24	47	1.02	16.50	0.23
*n* = 0	435	0.60	28	44	1.03	17.58	0

a) Specific surface area was calculated by BET modeling

b) Average cobalt particle size was calculated by using the Scherrer equation for the cobalt (111) peak in the XRD patterns

c) Average shell thickness was estimated by TEM analysis

d) *I*_D_/*I*_G_ was calculated from the ratio of the intensity of D‐band and G‐band in the Raman spectra

e) Co and Zn content determined by ICP‐OES

**Figure 4:**
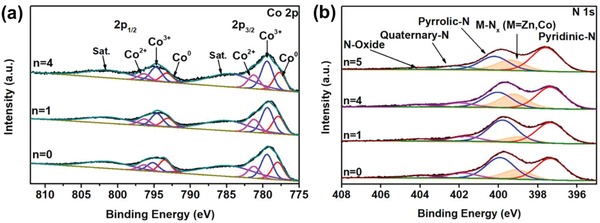
High‐resolution XPS spectra of a) Co 2p and b) N 1s for yolk‐shelled Zn*_n_*Co_5−_ *_n_*O*_x_*@carbon hollow capsules: n_Zn/Co_ = 5, n_Zn/Co_ = 4, n_Zn/Co_ = 1, and n_Zn/Co_ = 0.

Figure S9 of the Supporting Information illustrates the X‐ray photoelectron spectroscopy (XPS) survey spectra of yolk‐shell structured Zn*_n_*Co_5−_ *_n_*O*_x_*@carbon particles and their corresponding high‐resolution spectra of Co 2p and N 1s are shown in **Figure** 4a,b, respectively. The XPS survey spectrum of Zn_5_O*_x_*@carbon particles provides evidence for the existence of carbon (90.5 at%), nitrogen (3.8 at%), oxygen (5.3 at%), and zinc (0.4 at%), as shown in Table S1 (Supporting Information). After introduction of cobalt nanoparticles into carbon capsules, it is revealed that elemental zinc is not presented which is consistent with XRD and ICP‐OES results. The XRD patterns of cobalt‐based samples in Figure S6 of the Supporting Information shows no zinc species are found and only traceable zinc residuals are detected from ICP‐OES results in Table 1. In the high‐resolution Co 2p spectrum (Figure 4a), the Co 2p3/2 and Co 2p1/2 peaks are clearly found, which can be further deconvoluted into eight peaks at 777.7, 779.4, 781.3, 784.5, 793.2, 794.7, 796.4, and 801.2 eV. Among them, the characteristic metallic Co were observed with typical binding energies (BE) of 777.7 and 793.2 eV, assigned to Co 2p3/2 and Co 2p1/2 electrons of Co metal, respectively. The peaks at 781.3 and 796.4 eV are corresponded to the binding energies of the 2p3/2 orbitals of Co^2+^ species and the peaks at 779.4 and 794.7 eV can be ascribed to the 2p1/2 orbitals of Co^3+^ species.^50^ The peaks at 784.5 and 801.2 eV are satellite peaks that can be ascribed to the shakeup excitation of the high‐spin Co^2+^ ions.^54^ The presence of Co^2+^ and Co^3+^ may derive from the surface oxidation of the Co metal deposits exposing to air. This observation also revealed that Co atoms could partially have some interaction with the nitrogen or carbon atoms. Five peaks in the N 1s spectra at 397.3, 399.2, 400.0, 402.0, and 404.1 eV (Figure 4b) were attributed to pyridinic N, metal‐N*_x_* (M = Zn, Co), pyrrolic‐N, quaternary‐N, and N‐Oxide, respectively.^49^, ^55^ As presented in Table S2 of the Supporting Information, the ratio between graphitic‐N and total N atoms increased when the cobalt content was enhanced in the raw precursors, indicating that the higher the cobalt content, the higher the graphitization degree. It is worth‐mentioning that the percentage of Co‐N*_x_* in the total of nitrogen content of yolk‐shell structured Zn_4_Co_1_O*_x_*@carbon capsules was higher than that of the other four materials. It also reveals that such a coordination interaction between the cobalt species and nitrogen atoms is advantageous to improve the catalytic activity and prevent Co against serious aggregation in the hydrogenation process.^56^

Coating colloidal particles with polymer or carbon shell provides us a facile method to increase the structural complexity and functionality of colloidal particles. Silica dioxide (SiO_2_) materials have attracted great attention because of special properties such as excellent chemical and thermal stability, high surface area and controlled porous structure, and great importance in many fields including such as separation, sorption, controlled release, biosensing, and heterogeneous catalysis.^28^, ^29^, ^57^ Therefore, it is desirable to use SiO_2_ coating technique to prepare core@SiO_2_ structures. The synthetic strategy for the fabrication of porous hollow silica particles is similar to that of Zn*_n_*Co_5−_ *_n_*O*_x_*@carbon hollow capsules. Zn_4_Co_1_‐ZIF particles were synthesized first as hard templates and the versatile kinetics‐controlled coating method was used to deposit SiO_2_ layer on their surface. After hydrothermal treatment and the following calcination process, porous hollow silica particles can be achieved.

**Figure 5:**
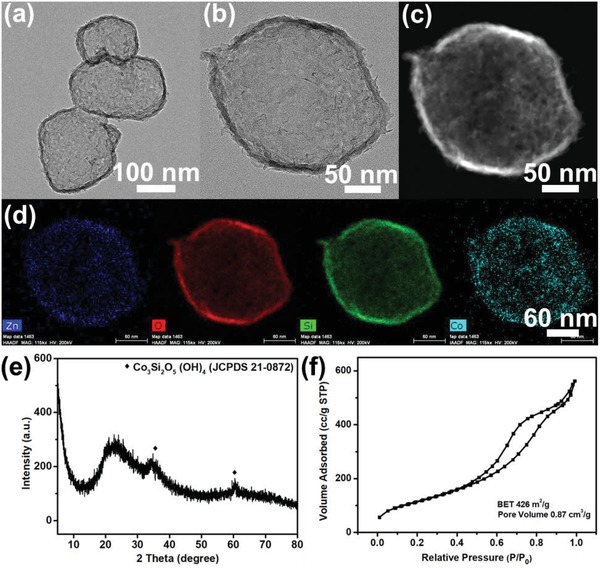
a,b) TEM images, c) HAADF image, d) STEM element mapping images, e) XRD pattern, and f) N_2_ adsorption–desorption isotherms of hollow structured silica composites (HSC).

As shown in Figure S10 of the Supporting Information, after coating SiO_2_ layer on the surface of ZIF particles and the following hydrothermal treatment, hollow‐structured SiO_2_ particle with shell thickness about 30 nm are obtained. **Figure** 5a,b show that after hydrothermal treatment and calcination in N_2_ atmosphere of Zn_4_Co_1_‐ZIF@SiO_2_, hollow structured silica composites (HSC) with decreased shell thickness about 13 nm are achieved. STEM and the corresponding elemental maps in Figure 5c,d illustrate that the Si and O atoms are dispersed uniformly throughout the whole framework. Additionally, elemental Zn and Co cannot only be found on the whole matrix but also within the hollow SiO_2_ shell. The XRD pattern of HSC is presented in Figure 5e. HSC particles display a broad diffraction peak at about 23°, which was the characteristic peak of SiO_2_. The remaining XRD peaks of HSC particles can be attributed to cobalt silicate hydroxide (JCPDS 21‐0871). The surface areas and pore volume of those as‐prepared nanoparticles were determined by nitrogen adsorption measurements, and the results are shown in Figure 5f. The nitrogen adsorption isotherms of the four samples are type IV with a distinct H3 hysteresis loop. The BET surface area and the pore volume obtained on the basis of the adsorption isotherm are 426 m^2^g^−1^ and 0.87 cm^3^ g^−1^, respectively. Additionally, TiO_2_ layer can be successfully coated on the surface of ZIF‐8, as shown in the TEM image of ZIF‐8@TiO_2_ of Figure S11a (Supporting Information). The TEM image in Figure S11b (Supporting Information) and HAADF image in Figure S11c (Supporting Information) present that after calcination in air, hollow structured zinc titanium composites can be achieved. The elemental mapping results in Figure S11d (Supporting Information) suggest zinc, titanium, and oxygen atoms are dispersed uniformly throughout the whole framework.

**Figure 6:**
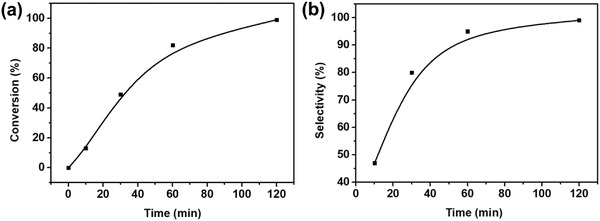
Reaction profiles of a) catalytic conversion and b) selectivity for nitrobenzene over Zn_4_Co_1_O*_x_*@carbon hollow capsules.

To evaluate the catalytic performance of the as‐prepared Zn*_n_*Co_5−_ *_n_*O*_x_*@carbon hollow capsules, the hydrogenation of nitrobenzene to aniline was performed at 70 °C under H_2_ (5 MPa) over a period of 2 h. As shown in **Table** 2, no catalytic activity was found when Zn_5_O*_x_*@carbon particles (Table 2, entry 1) were used for hydrogenation of nitrobenzene within 2 h, demonstrating Co was the active site for the hydrogenation reaction under the investigated conditions. Zn_4_Co_1_O*_x_*@carbon particles proved to be a superb catalyst for the hydrogenation of nitroarenes (Table 2, entry 2). Both nitrobenzene conversion and selectivity on Zn_4_Co_1_O*_x_*@carbon particles increases gradually with reaction time and reaches 99% at 2 h (**Figure** 6). In comparison with Zn_4_Co_1_O*_x_*@carbon particles, Zn_1_Co_4_O*_x_*@carbon particles (conversion 60%, Table 2, entry 3) and Co_5_O*_x_*@carbon particles (conversion 59%, Table 2, entry 4) show markedly lower hydrogenation efficiency and selectivity in the hydrogenation of nitrobenzene. The turnover frequency (TOF) of Zn_4_Co_1_O*_x_*@carbon particles (169 h^−1^; Table S3, Supporting Information) is about 4.3‐fold that of Zn_1_Co_4_O*_x_*@carbon particles (39 h^−1^; Table S3, Supporting Information) and Co_5_O*_x_*@carbon particles (39 h^−1^; Table S3, Supporting Information). Furthermore, optimal conversion and selectivity of the Zn*_n_*Co_5−_ *_n_*O*_x_*@carbon particles was found using smaller molar ratios of S/C (Table 2; entry 5–6). These results indicated that small and highly dispersed cobalt nanoparticles are vital for achieving high catalytic activity. In general, several factors including particle composition, size, and the nature of the support materials are of great importance for catalysts performance.^58^, ^59^ As to this novel yolk‐shell structured Zn_4_Co_1_O*_x_*@carbon material, the high hydrogenation catalytic conversion and selectivity is ascribed to the hollow carbon morphology, which facilitates the mass transportation of nitrobenezene, enhancing the collision frequency and adsorption of nitrobenzene on the surface of cobalt nanoparticles. In addition, the relatively small cobalt nanoparticles, high dispersion of cobalt nanoparticles, large specific surface areas, and high proportion of Co‐N*_x_* in the total nitrogen content of yolk‐shell structured Zn_4_Co_1_O*_x_*@carbon particles among all the prepared samples also lend additional promotion of the catalytic hydrogenation reaction. Therefore, the superior conversion and selectivity of nitrobenzene over Zn_4_Co_1_O*_x_*@carbon particles can be attributed to particular set of properties and characteristics displayed by this carbon capsules.

**Table 2:** Hydrogenation of nitrobenzene catalyzed by Zn*_n_*Co_5−_ *_n_*O*_x_*@carbon hollow capsulesa)

Entry	S/C	Catalyst Zn*_n_*Co_5−_ *_n_*O*_x_*@carbon	Conversion [%]	Selectivity [%]
1	10	*n* = 5	0	0
2	10	*n* = 4	>99	>99
3	10	*n* = 1	60	86
4	10	*n* = 0	59	81
5	25	*n* = 4	88	91
6	40	*n* = 4	34	87

a) Reaction conditions: nitrobenzene 0.2 mmol, 70 °C; 2 h, 5 MPa H_2_, 1 mL THF, 100 µL H_2_O.

To explore a more general application of Zn_4_Co_1_O*_x_*@carbon particles, various nitroarenes with different electron‐donating groups (such as methyl and hydroxyl groups) and electron‐withdrawing groups (such as ester and ketone groups) at different positions, with respect to the benzene rings, were investigated under the same catalytic conditions. Beller and co‐workers reported the possible mechanism of hydrogenation of nitrobenzene to aniline by using cobalt catalysts.^60^ It was proposed that the pyridinic N content is important for the catalyst activity and the active site was derived from Co–N–C network. Additionally, it was postulated that this kind of hydrogenation proceed through a heterolytic activation of dihydrogen. The nitro group from nitro compounds is preferably adsorbed on the catalyst surface rather than functional groups and the high contribution of Co‐N species is beneficial for the H_2_ dissociation, thus leading to a high selectivity toward various nitrobenzene. As shown in **Table** 3, Zn_4_Co_1_O*_x_*@carbon particles show high activity and selectivity for nitroarene hydrogenation toward their corresponding anilines. When chlorine groups were introduced on the nitrobenzene, the chloroanilines can be obtained in excellent yields (99%) with superior selectivity (Table 3, entry 1). For hydrogenation of nitrobenzene with electron donating substituents, such as para‐, ortho‐, and metanitrotoluene, nearly 99% conversion and 99% selectivity can be reached by using Zn_4_Co_1_O*_x_*@carbon particles, indicating that the effect of space steric hindrance from the methyl group had little effect on the hydrogenation of the nitro group (Table 3, entries 2–4). Particularly, the nitroarenes electron‐withdrawing groups (such as ester and ketone groups) have also been successfully reduced to their corresponding aniline products with excellent conversion and selectivity (Table 3, entries 5–8), highlighting the admirable chemoselectivity of the transitional metal catalysts and the remarkable advantage compared with noble metal catalysts.

**Table 3:**
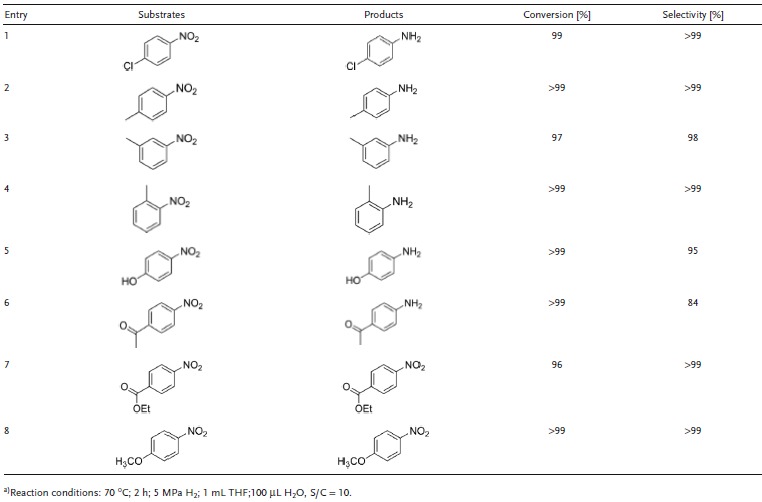
Catalytic performance of Zn_4_Co_1_O*_x_*@carbon particles in the hydrogenation of various nitro compounds^a)^

The recyclability of Zn_4_Co_1_O*_x_*@carbon particles was investigated using nitrobenzene hydrogenation as model reaction (Figure S12, Supporting Information). The solid Zn_4_Co_1_O*_x_*@carbon catalyst can be easily recovered by centrifugation after reaction and was successfully reused for eight cycles without any obvious loss of catalytic activity for the hydrogenation of nitrobenzene. These results indicate the excellent stability of Zn_4_Co_1_O*_x_*@carbon catalyst within this reaction, which can also be presumed to extend to the hydrogenation of other nitroarenes.

**Figure 7:**
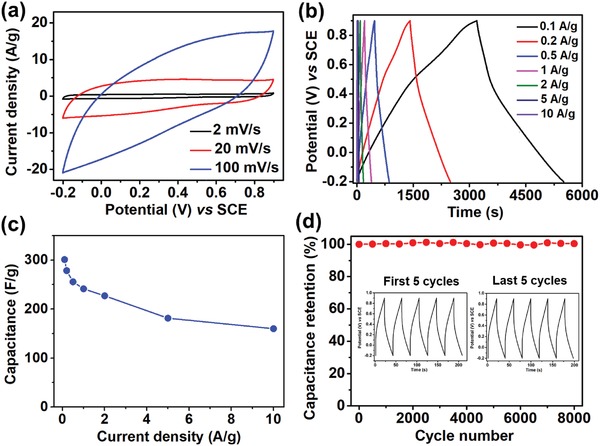
a) CV curves of Zn_4_Co_1_O*_x_*@carbon at the scan rate of 2, 20, and 100 mV s^−1^ in 1 m H_2_SO_4_ aqueous solution. b) GCD curves of the Zn_4_Co_1_O*_x_*@carbon electrode at different current densities. c) Specific capacitances of the Zn_4_Co_1_O*_x_*@carbon electrode as a function of scan rate. d) Cycling stability of the Zn_4_Co_1_O*_x_*@carbon electrode at a current of 5 A g^−1^. Inset is the first and last five charge/discharge cycles of Zn_4_Co_1_O*_x_*@carbon.

Owing to the outstanding properties of Zn_4_Co_1_O*_x_*@carbon sample such as high surface areas, short mass diffusion, and transport resistance, it may also exhibit excellent performance in other structure determined application.^26^ Therefore, we expanded our study on electrochemical properties for further stability investigation.The cyclic voltammetry (CV) curves of Zn_4_Co_1_O*_x_*@carbon electrode at various scan rates of 2–100 mV s^−1^ are shown in **Figure** 7a. The quasirectangular CV shape without any redox peaks indicates a double‐layer capacitive behavior. Quasirectangular shape remained even at a high scan rate of 100 mV s^−1^, indicating fast charge‐transfer characteristics. This was further supported by the low charge‐transfer resistance value *R*_ct_ of ≈6 Ω (Figure S13, Supporting Information). The galvanostatic charge–discharge (GCD) curves (Figure 7b) at different current densities display a nearly triangular shape, implying considerable electrochemical reversibility. The specific capacitance of the Zn_4_Co_1_O*_x_*@carbon electrode calculated from the GCD curves (Figure 7c) is 301 F g^−1^ at 0.1 A g^−1^. Comparing this value to that obtained from Zn_1_Co_4_‐ZIF, a material with increased cobalt content, revealed a lower capacitance of ≈153 F g^−1^ (as shown in Figure S14 of the Supporting Information). This is most likely due to a larger particle surface area for the Zn_4_Co_1_O*_x_*@carbon material, in addition to increased pore volume and higher nitrogen‐doping content than Zn_1_Co_4_O*_x_*@carbon particles.^61^, ^62^ In addition, the Zn_4_Co_1_O*_x_*@carbon electrode exhibits excellent cycling stability with ≈100% capacitance retention after 8000 charge/discharge cycles (Figure 7d).

## 3 Conclusions

In summary, we have developed a highly efficient Co@carbon yolk shell material by direct pyrolysis of Zn/Co‐ZIF@polymer precursors. The resultant catalysts benefit from a high surface area, small and well dispersed cobalt particles within a nitrogen‐doped hollow carbon shell. In addition, agglomeration of catalytically active Co nanoparticles can be prevented by the formed yolk‐shell structures and mass transfer can be further promoted by the presence of mesopores throughout the carbon structure. The strong interaction between the cobalt nanoparticles and the surrounding carbon shell provide positive synergistic effects, similar to well‐known support/active phase interactions. Furthermore, these interactions are greatly enhanced by the presence of Zn within the Co structure, that also serves to better disperse the Co particles. These characters endow the unique properties to our yolk‐shell structured nanoreactor that goes on to display superior catalytic activities (>99% selectivity and >99% conversion), stability, and reusability in hydrogenation of nitrobenzene at mild conditions. The simple and low‐cost method discussed in this work opens up new prospects for the applications of multiple functionalized yolk‐shell nanoparticles as efficient nanoreactors for various applications.

## 4 Experimental Section

*Chemicals*: The following chemicals were purchased from Sigma Aldrich (unless otherwise stated) and used as received, without further purification: aqueous ammonia (25% solution), formaldehyde (37% solution), 3‐aminophenol (99%), methanol (99%), (PVP, *M*_w_ ≈ 50 000), ethanol (95–100%), zinc nitrate hexahydrate (Zn(NO_3_)_2_·6H_2_O), cobalt nitrate hexahydrate (Co(NO_3_)_2_·6H_2_O), and 2‐methylimidazole. Washing was achieved with ultrapure water produced on site and reagent grade ethanol. Ultrapure water was used for solution preparations.

*Synthesis of ZIF‐8, ZIF‐67, and Bimetallic ZIFs*: Synthesis of ZIF‐8—Zn(NO_3_)_2_·6H_2_O (0.89 g) was dissolved in 30 mL of methanol containing 0.5 g PVP (*M*_W_ 50 000) to form a solution. 20 mL of methanol containing 2‐methylimidazole (1.97 g) was poured into the Zn(NO_3_)_2_ solution. The mixture was subsequently kept at room temperature without stirring for 24 h. The resulting white precipitates were collected by centrifuging, washed with methanol three times, and finally dried at 100 °C overnight.

Synthesis of ZIF‐67—Co(NO_3_)_2_·6H_2_O (0.87 g) was dissolved in 30 mL of methanol containing 0.5 g PVP (*M*_W_ 50 000) to form a solution. 20 mL of methanol containing 2‐methylimidazole (1.97 g) was poured into the Co(NO_3_)_2_ solution. The mixture was kept at room temperature without stirring for 24 h. The resulting purple precipitates were collected by centrifuging, washed with methanol three times, and finally dried at 100 °C overnight.

Synthesis of bimetallic ZIFs (Zn*_n_*Co_5_ *_−n_‐*ZIF)*—*Zn(NO_3_)_2_·6H_2_O (0.18 g) and Co(NO_3_)_2_·6H_2_O (0.70 g) were dissolved in 30 mL of methanol containing 0.5 g PVP (*M*_W_ 50 000) to form a solution. 20 mL of methanol containing 2‐methylimidazole (1.97 g) was poured into the mixture of Zn(NO_3_)_2_ and Co(NO_3_)_2_ solution. The mixture was kept at room temperature without stirring for 24 h. The resulting precipitates were collected by centrifuging, washed with methanol three times, and finally dried at 100 °C overnight, resulting in the so‐called Zn_1_Co_4_‐ZIF. Similarly, for the synthesis of Zn_4_Co_1_‐ZIF, the procedures were carried out except that the Zn/Co molar ratio was 4, respectively.

*Synthesis of Yolk‐Shelled Zn_n_Co*_5_ *_−n_O_x_@Polymer*: In a typical synthesis, ammonia (NH_4_OH, 0.2 mL, 25 wt%) was added in a mixture of water (20 mL) and ethanol (8 mL) and stirred at room temperature for 0.5 h, followed by addition of 0.1 g ZIF particles. After stirring for 0.5 h, 3‐aminophenol (0.12 g) was added into that suspension. After stirring for an additional 30 min, formaldehyde (0.168 mL) was added. The mixture was stirred for 24 h at room temperature and subsequently heated for 24 h at 140 °C under static conditions in a Teflon‐lined autoclave. The solid products were recovered by centrifugation and dried at 100 °C for 24 h. The remaining materials were named as yolk‐shelled Zn*_n_*Co_5−_ *_n_*O*_x_*@polymer.

*Synthesis of Zn_n_Co*_5_ *_−n_O_x_@Carbon Hollow Capsules*: Zn*_n_*Co_5−_ *_n_*O*_x_*@polymer nanoparticles were carbonized in flowing N_2_ in a tube furnace using a heating rate of 1 °C min^−1^ up to 350 °C, dwell for 2 h, and resuming heating rate at 1 °C min^−1^ up to 700 °C and dwell for 4 h. The remaining materials were named as Zn_n_Co_5−n_O*_x_*@carbon.

*Catalytic Hydrogenation of Nitrobenzene*: A desired amount of solid catalyst was added in an ampoule tube, followed by the addition of nitrobenzene and 1 mL of tetrahydrofuran (THF) and 100 µL H_2_O. The ampoule tube was loaded into a stainless‐steel autoclave (300 mL) with a thermocouple probed detector. After purging with hydrogen for four times, the reactor was heated to 70 °C with vigorous stirring and then pressure was adjusted to 5 MPa. After reaction for 2 h, the solid catalyst was separated by centrifugation and the filtrate was collected, diluted with THF and analyzed by a SHIMADZU GC‐2130 equipped with an InertCap 5 capillary column (30 m × 0.32 mm × 0.25 µm).

For recycle experiments, the catalysts were separated by centrifugation after reaction, washed twice with ethanol before a further two washes with THF. Finally, the residual catalysts were transferred back to the reactor without drying for the next catalytic run under standard reaction conditions.

*Materials Characterization*: The sample morphology was characterized by using an SEM (FEI Verios XHR) and TEM (JEOL EM‐2100). High Angle Annular Dark Field STEM (HAADF‐STEM) imaging and element mapping were carried out using an FEI Titan G2 80–200 TEM/STEM with ChemiSTEM Technology operating at 200 kV. The elemental maps were obtained by energy dispersive X‐ray (EDX) spectroscopy using the Super‐X detector on the Titan with a probe size ≈1 nm and a probe current of ≈0.4 nA. Powder XRD analysis was performed on an X‐ray diffractometer (Bruker D8 Advance) using Cu Kα radiation source (40 kV and 30 mA). The BET specific surface area and single‐point pore volume were obtained from nitrogen adsorption isotherms measured at −196 °C using a nitrogen sorption instrument (Micromeritics TriStar II Surface Area and Porosity Analyzer). Prior to nitrogen adsorption measurements, the samples were degassed at 250 °C overnight. Cobalt content in Zn*_n_*Co_5−_ *_n_*O*_x_*@carbon particles was determined by Inductively Coupled Plasma Optical Emission Spectrometry (ICP‐OES).

## Conflict of Interest

The authors declare no conflict of interest.

## Supporting information

Supplementary
