# Supplementary material for: Enhanced Hydrogenation Performance over Hollow Structured Co‐CoOx@N‐C Capsules
Source: Adv Sci (Weinh). 2019 Sep 3;6(22):1900807. doi: 10.1002/advs.201900807 (PMC6865004; doi:10.1002/advs.201900807)
Supplement: Supplementary file 1 — Supplementary [file ADVS-6-1900807-s001.pdf]

Copyright WILEY-VCH Verlag GmbH & Co. KGaA, 69469 Weinheim, Germany, 2019.

Supporting Information

for *Adv. Sci.,* DOI: 10.1002/advs.201900807

Enhanced Hydrogenation Performance over Hollow Structured Co-CoO*x*@N-C Capsules

*Hao Tian, Xiaoyan Liu, Liubing Dong, Xiaomin Ren, Hao Liu, Cameron Alexander Hurd Price, Ying Li, Guoxiu Wang, Qihua Yang, and Jian Liu**

Supporting Information

Enhanced Hydrogenation Performance over Hollow Structured Co-CoO*x*@N-C Capsules

*Hao Tian, Xiaoyan Liu, Liubing Dong, Xiaomin Ren, Hao Liu, Cameron Alexander Hurd Price, Ying Li, Guoxiu Wang, Qihua Yang and Jian Liu**

[a] Dr H. Tian, X. Liu, X. Ren, C. Price, Prof. Q. H. Yang, Prof. J. Liu
State Key Laboratory of Catalysis, iChEM, Dalian Institute of Chemical Physics
Chinese Academy of Sciences
457 Zhongshan Road, Dalian 116023, China
E-mail: jianliu@dicp.ac.cn

[b] Dr H. Tian, Prof. J. Liu
Department of Chemical Engineering, Curtin University
Perth, WA 6845, Australia

[c] Dr H. Tian, Dr L. Dong, Prof. H. Liu, Prof. G. Wang
Centre for Clean Energy Technology, School of Mathematical and Physical Sciences, Faculty of Science, University of Technology Sydney, Broadway, Sydney, NSW 2007, Australia

[d] Prof. H. Liu
Joint International Laboratory on Environmental and Energy Frontier Materials, School of Environmental and Chemical Engineering, Shanghai University, Shanghai, 200444, China.

[e] C. Price, Prof. J. Liu
DICP-Surrey Joint Centre for Future Materials, Department of Chemical and Process Engineering, and Advanced Technology Institute, University of Surrey, Guilford, Surrey, GU2 7XH, UK
E-mail: jian.liu@surrey.ac.uk

[f] X. Liu, Prof. Y. Li
Institute of Industrial Catalysis, Zhejiang University of Technology, 18 Hangzhou Chaowang Road 310032, China.

Turnover frequency (TOF) calculations

Turnover frequencies (TOF) were measured by using a desired amount of solid yolk-shell structured $Zn_nCo_{5-n}O_x$@carbon catalyst without otherwise altering the catalysis reaction conditions. The results were collected when nitrobenzene was consumed over 10 mins. In a typical experiment, 40 µl of nitrobenzene, 1 mL of THF and a desired amount of solid catalyst of catalyst were added into an autoclave reactor (300 mL), which was sealed and purged with H_2 several times then heated for 10 min at 70 °C under 5 MPa H_2 with stirring at 400 RPM. The TOFs of these three catalysts were calculated based on their total Co content.

$$TOF=n_0C/tn_{cat}$$

Where n_0 is the initial number of moles of substrate, C is the percentage conversion of the substrate at a reaction time of t (h), and n_{cat} is number of moles of Co in the catalyst.

Electrochemical measurements

Electrodes were prepared by mixing the $Zn_nCo_{5-n}O_x$@carbon powder, carbon black and polyvinylidene fluoride at a weight ratio of 7:2:1 in 1-methyl-2-pyrrolidone solvent, and then coating the mixture onto stainless steel net. Cyclic voltammetry (CV), Galvanostatic charge-discharge (GCD) and Electrochemical impedance spectroscopy (EIS, 100 kHz to 10 mHz with an amplitude of 5 mV) techniques were applied to evaluate electrochemical behaviors of the electrode in a three-electrode system, in which our electrode, saturated calomel electrode (SCE) and Pt sheet were used as working electrode, reference electrode and counter electrode, respectively. 1 M H_2SO_4 aqueous solution served as the electrolyte. Above tests was performed in a Biologic VMP3 electrochemical station. Cycling stability was estimated using GCD test at a current density of 5 A/g.

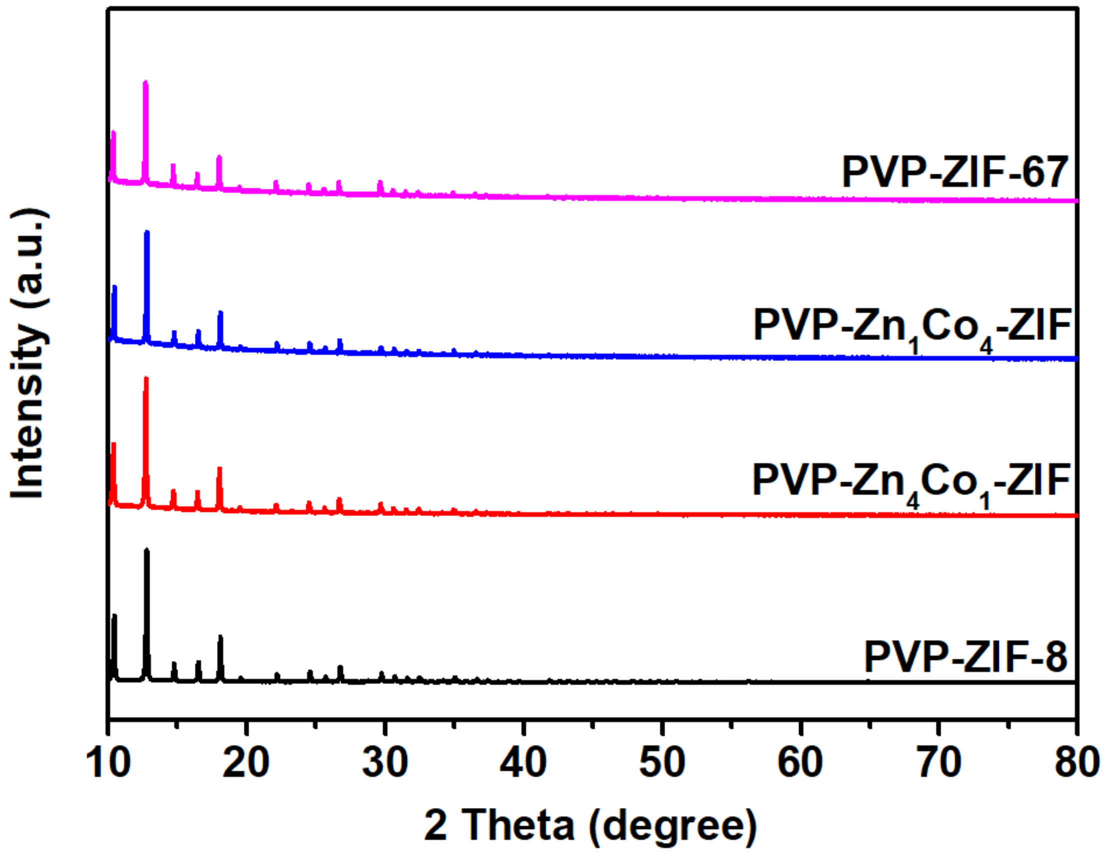

Figure S1. XRD patterns of ZIF-8, ZIF-67 and bimetallic ZIFs (Zn_nCo_{5-n}-ZIF) nanoparticles. $n_{Zn/Co}$=4 and 1.

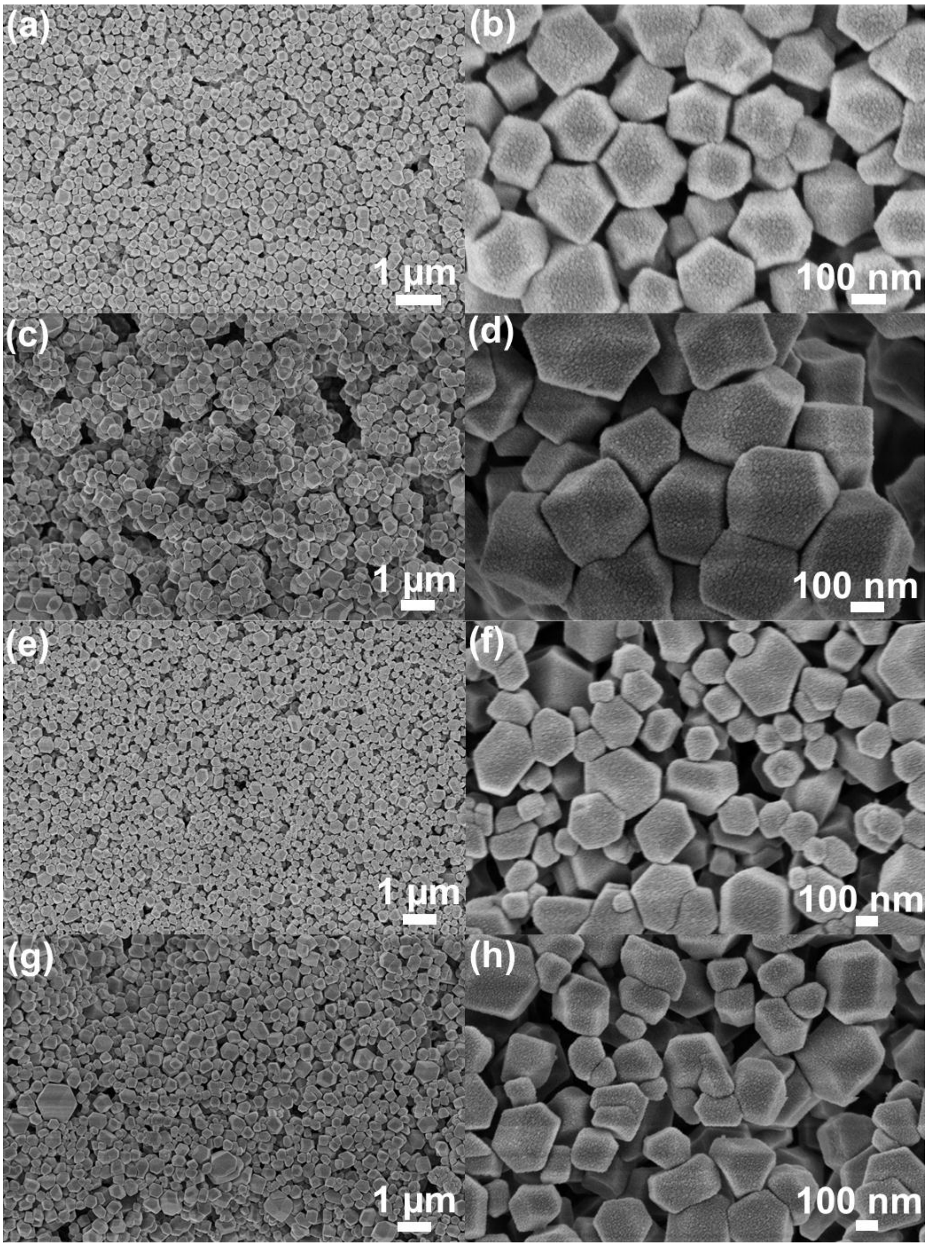

Figure S2. SEM images of ZIF-8 (a) (b), bimetallic Zn_nCo_{5-n}-ZIFs nanoparticles ($n_{Zn/Co}$=4 (c) (d), and $n_{Zn/Co}$=1 (e) (f)), ZIF-67 (g) (h).

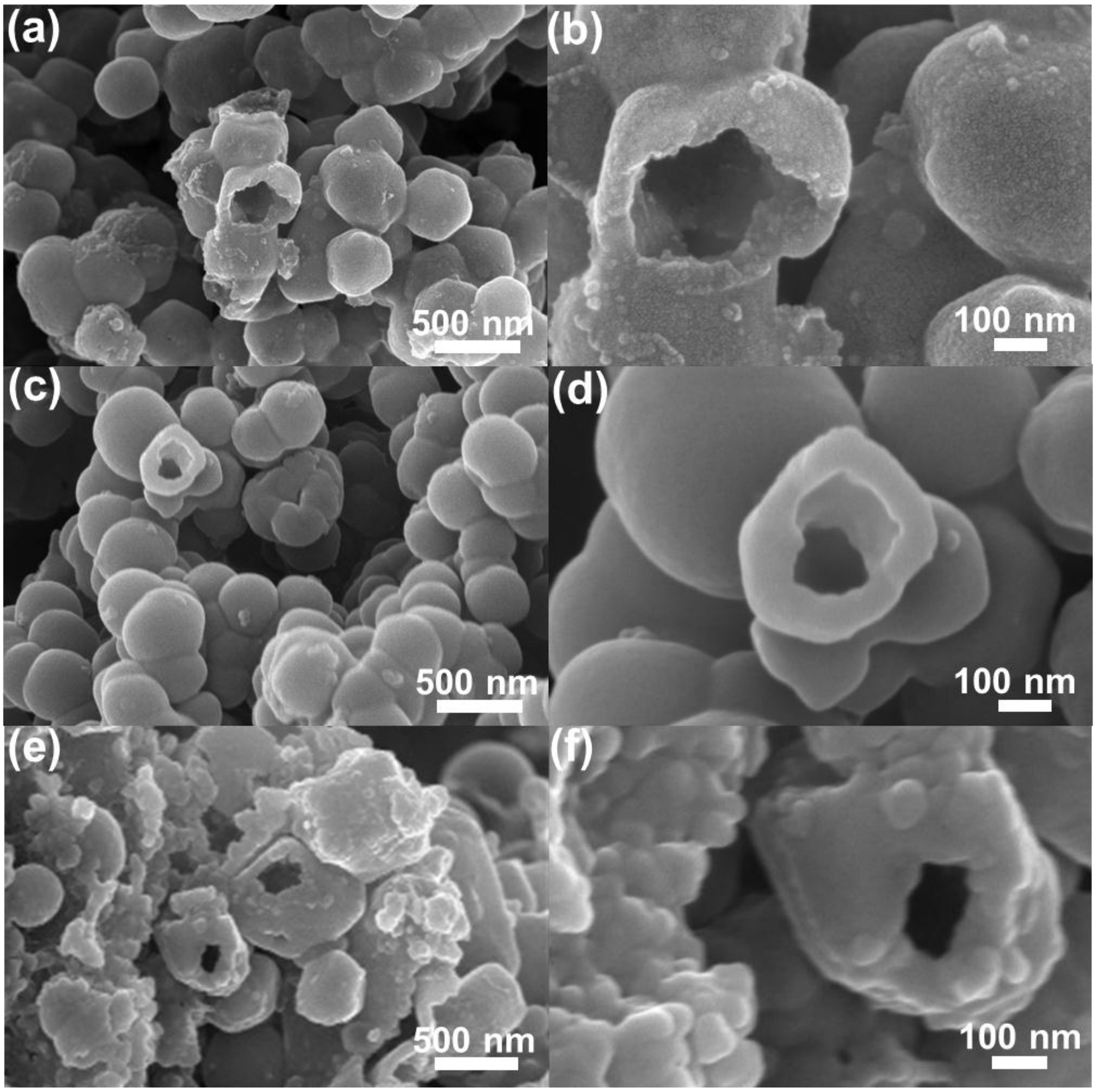

Figure S3. SEM images of yolk-shelled $Zn_nCo_{5-n}O_x$@thin carbon hollow capsules. (a) (b) $n_{Zn/Co}$=4, (c) (d) $n_{Zn/Co}$=5 and (e) (f) $n_{Zn/Co}$=1.

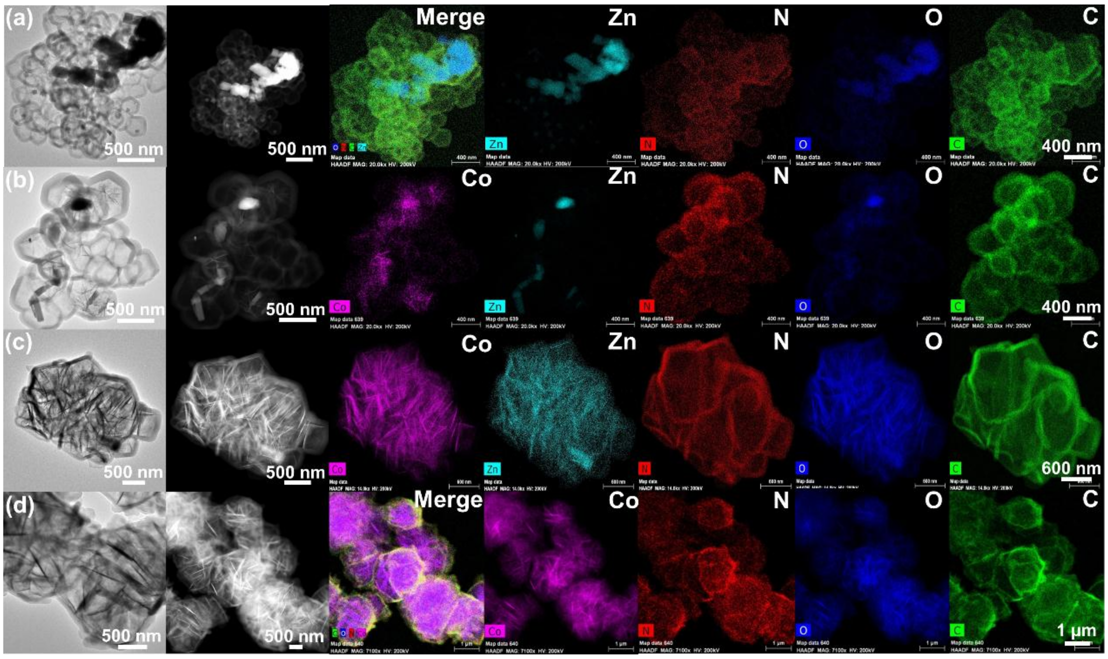

Figure S4. TEM images, HAADF image, STEM element mapping images of yolk-shelled $Zn_nCo_{5-n}O_x$@thin polymer. (a) $n_{Zn/Co}$=5, (b) $n_{Zn/Co}$=4, (c) $n_{Zn/Co}$=1, (d) $n_{Zn/Co}$=0.

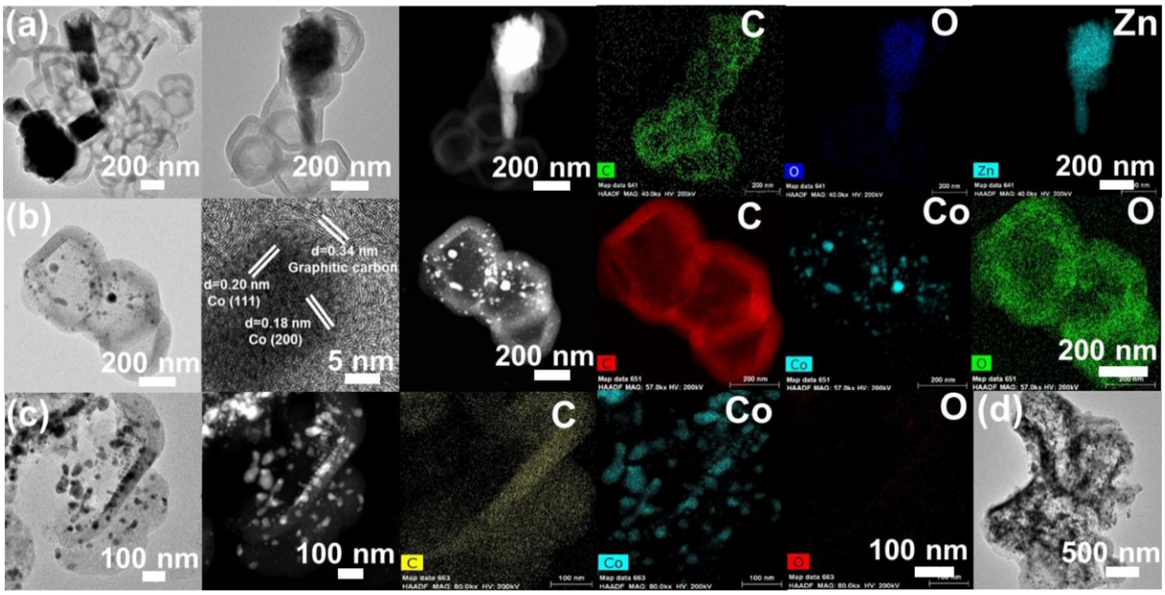

Figure S5. TEM images, HAADF image, STEM element mapping images of yolk-shelled $Zn_nCo_{5-n}O_x$@thin carbon hollow capsules. (a) $n_{Zn/Co}$=5, (b) $n_{Zn/Co}$=4, (c) $n_{Zn/Co}$=1, (d) $n_{Zn/Co}$=0.

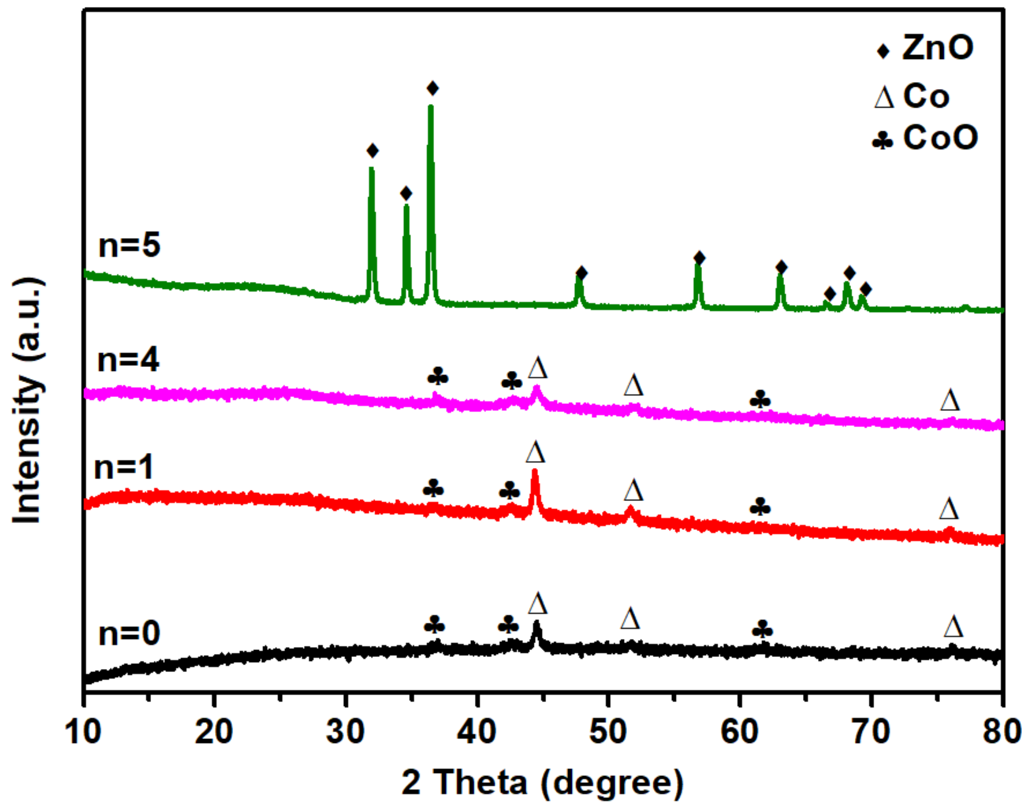

Figure S6. XRD patterns of yolk-shelled $Zn_nCo_{5-n}O_x$@carbon hollow capsules: n=5, n=4, n=1, n=0.

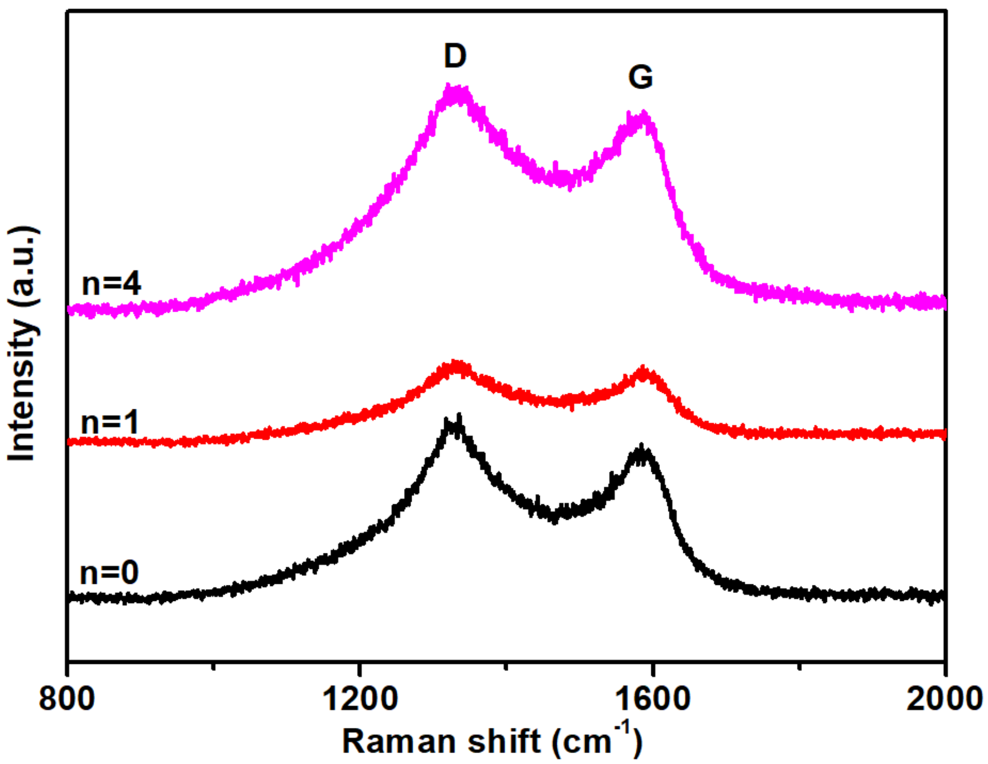

Figure S7. Raman spectra of yolk-shelled $Zn_{n}Co_{5-n}O_{x}$@carbon hollow capsules: n=4, n=1, n=0.

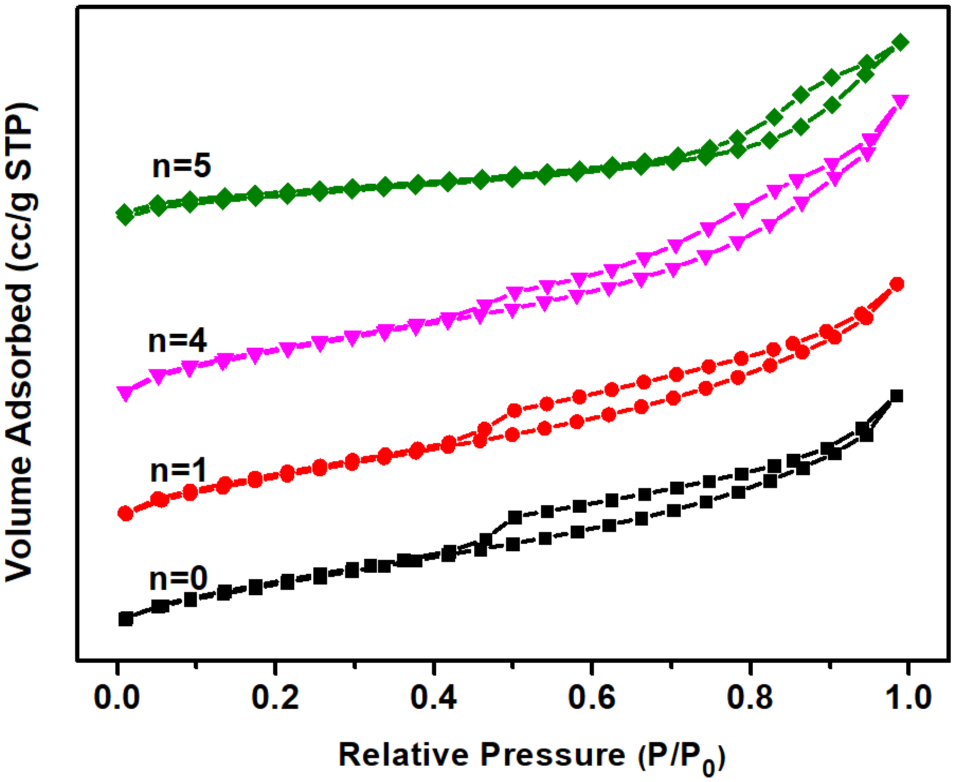

Figure S8. N_2 adsorption–desorption isotherms of yolk-shelled $Zn_nCo_{5-n}O_x$@carbon hollow capsules: n=5, n=4, n=1, n=0.

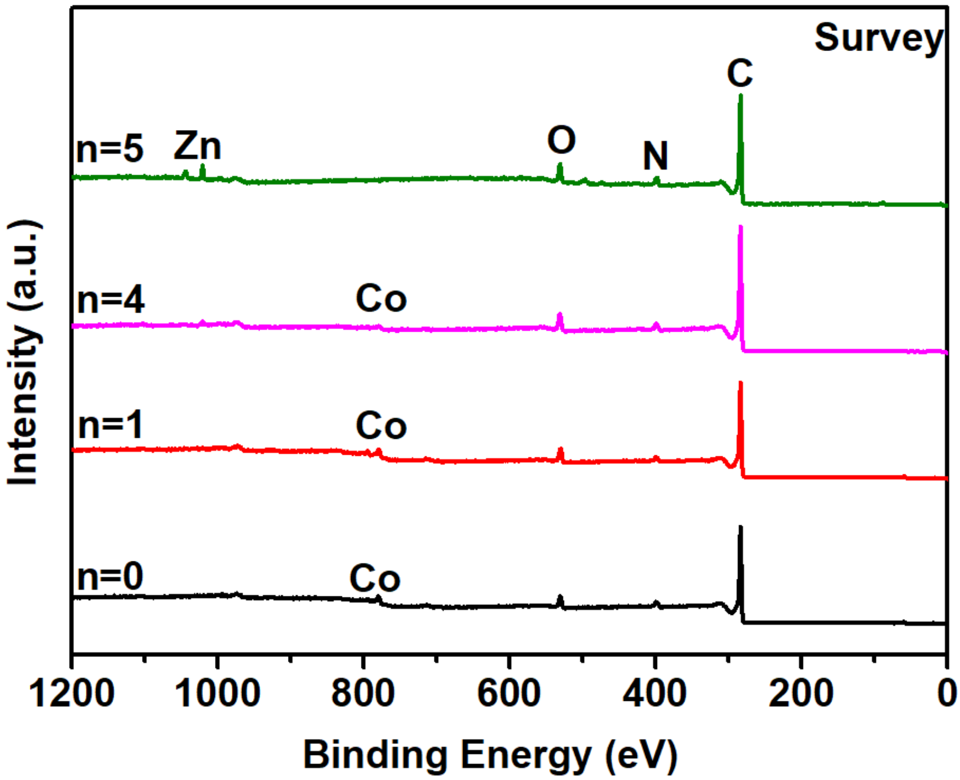

Figure S9. Survey spectra of yolk-shelled $Zn_nCo_{5-n}O_x$@carbon hollow capsules: n=5, n=4, n=1, n=0.

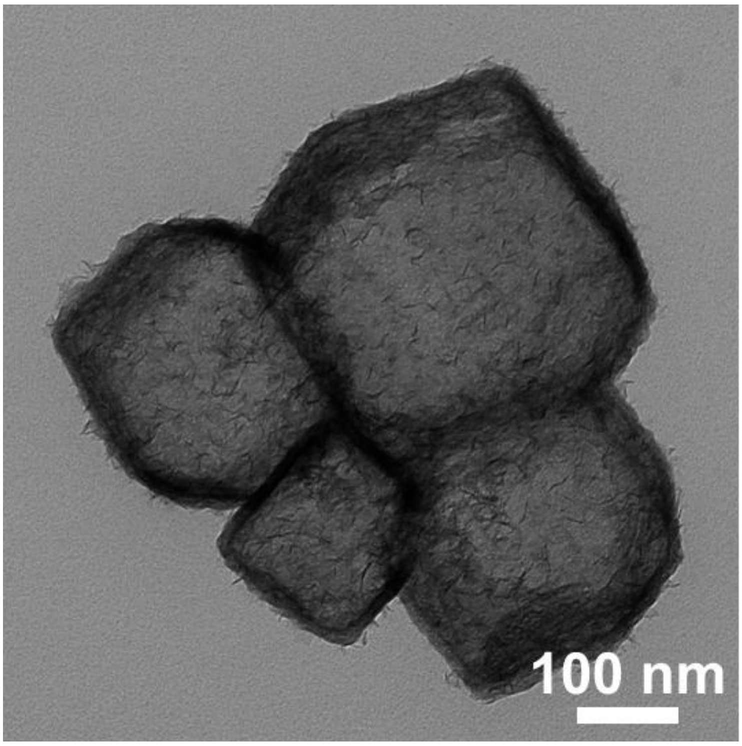

Figure S10. TEM image of Zn_4Co_1-ZIF@SiO_2-after hydrothermal.

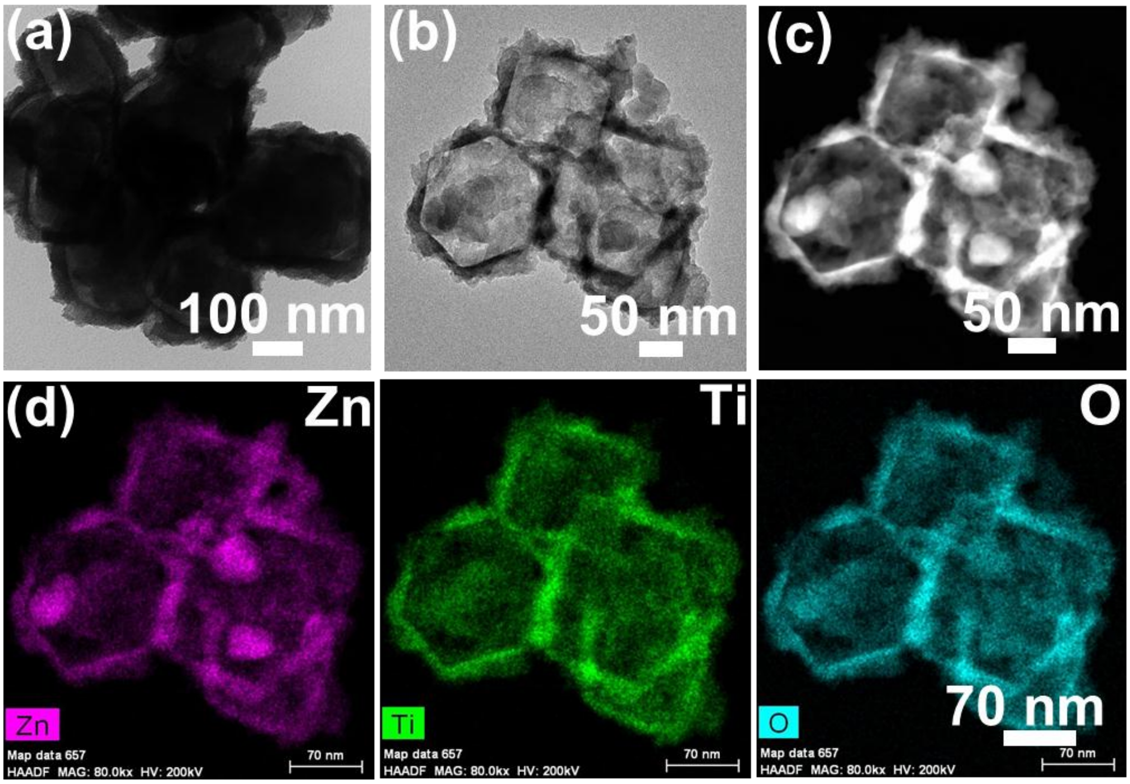

Figure S11. (a) TEM image of ZIF-8@TiO_2; (b) TEM image, (c) HAADF image and (d) element mapping images of ZIF-8@TiO_2 after calcination in air.

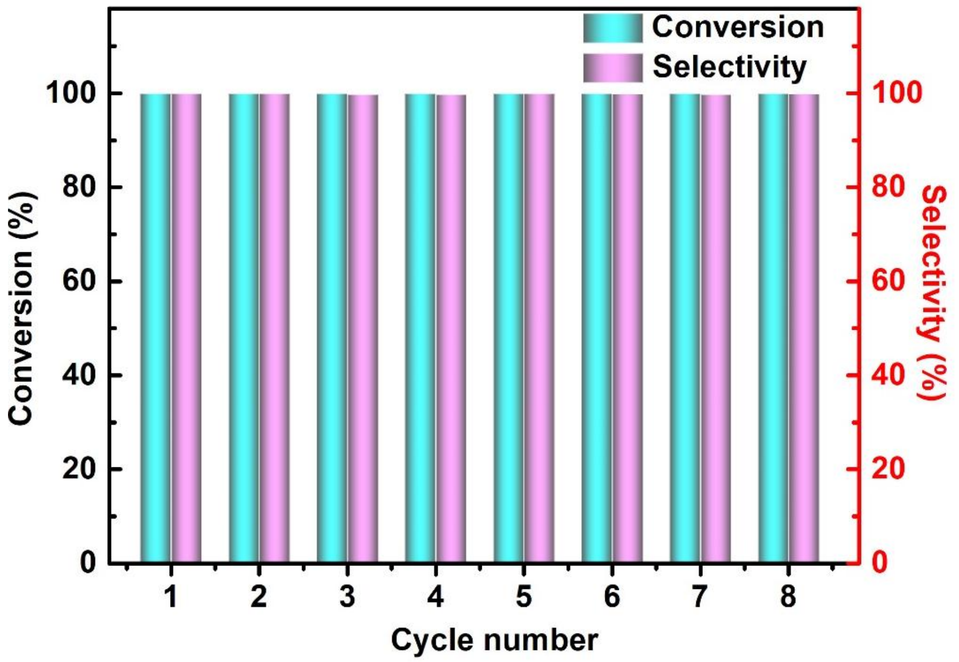

Figure S12. The reusability of Zn_4Co_1Ox@carbon particles.

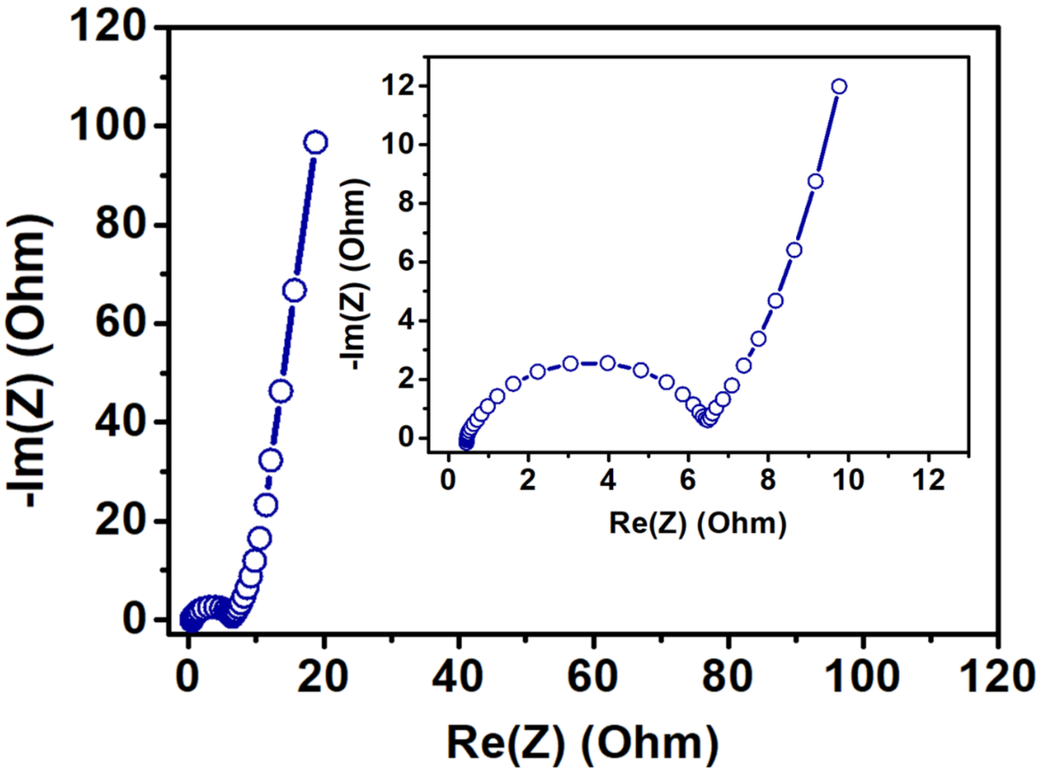

Figure S13. EIS plot of the $Zn_4Co_1O_x$@carbon electrode.

The EIS measurements were carried out in the frequency range from 100 kHz to 10 mHz. The curve consists of a semicircle in high frequency region and a straight line in low frequency region. The

semicircle diameter reflects the charge transfer resistance, while the slope of straight line indicates the ion diffusion resistance. The $Zn_4Co_1O_x$@carbon exhibits a smaller semicircle diameter only ~6 Ω and large slope, verifying a fast charge transfer kinetics at the electrode/electrolyte interface, which is helpful for the superior rate capability.

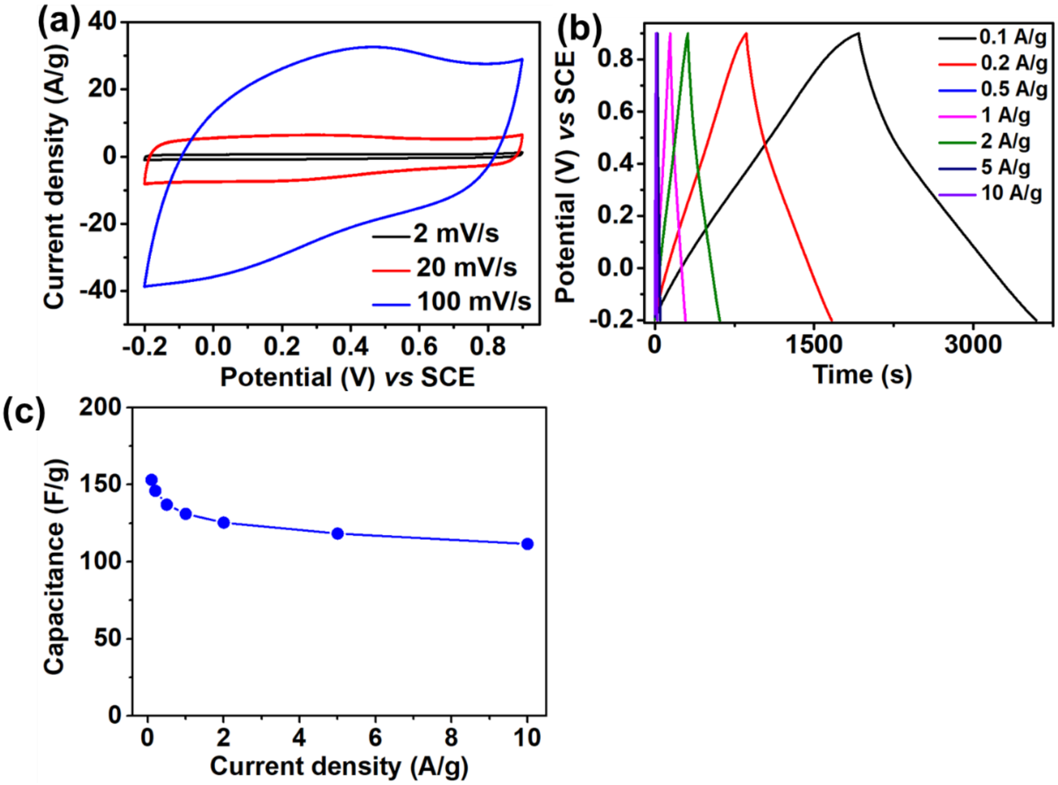

Figure S14. (a) CV curves of Zn_1Co_4Ox@carbon at the scan rate of 2, 20 and 100 mV s^{-1} in 1 M H_2SO_4 aqueous solution. (b) Galvanostatic charge-discharge curves of the $Zn_1Co_4O_x$@carbon electrode at different current densities. (c) Specific capacitances of the Zn_1Co_4Ox@carbon electrode as a function of scan rate.

Table S1. The chemical compositions of $Zn_nCo_{5-n}O_x$@carbon capsules obtained by XPS characterization.

$Zn_nCo_{5-n}O_x$@carbon	C (at.%)	N (at.%)	O (at.%)	Co (at.%)	Zn (at.%)
n=5	90.5	3.8	5.3	-	0.4
n=4	92.6	2.8	4.4	0.2	
n=1	92.5	2.5	4.6	0.4	
n=0	92.8	2.9	3.9	0.4	

Table S2. The atomic percentage of Pyridinic N, Pyrrolic N, Quaternary N and N-Oxide of $Zn_nCo_{5-n}O_x$@carbon capsules obtained by their corresponding high−resolution N 1s XPS spectrum.

$Zn_nCo_{5-n}O_x$@carbon	Pyridinic N (at.%)	Zn, Co-N_x (at.%)	Pyrrolic N (at.%)	Quaternary N (at.%)	N-Oxide (at.%)
n=5	50.3	11.1	29.0	7.6	2.0
n=4	42.6	14.0	30.1	8.2	5.1
n=1	37.3	10.2	39.6	8.9	4.0
n=0	40.4	8.4	39.4	9.4	2.4

Table S3. Hydrogenation of nitrobenzene catalysed by $Zn_nCo_{5-n}O_x$@carbon hollow capsules

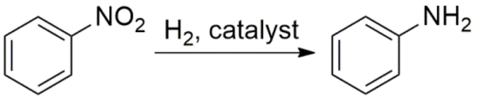

Entry	Catalyst $Zn_nCo_{5-n}O_x$@carbon	10 min		
		Conversion (%)	Selectivity (%)	TOF (h^{-1})
1[a]	n=5	~0	~0	0
2[a]	n=4	13	47	169
3[a]	n=1	3	34	39
4[a]	n=0	3	30	39

[a] Reaction conditions: 70 °C; 10 min; 5 MPa H_2; 1 mL THF;100 µLH_2O.
